# Vaping-induced acute epiglottitis: a case report

**DOI:** 10.1186/s12245-023-00532-x

**Published:** 2023-09-05

**Authors:** Amir Khorrami, Mohammad Ali Khorrami, Heitham Gheriani

**Affiliations:** 1 Faculty of Medicine, University of British Columbia, Vancouver, BC Canada; 2 Department of Surgery Division of Otolaryngology, University of British Columbia, Vancouver, BC Canada

**Keywords:** Epiglottitis, Electronic cigarettes, Vaping, Inflammatory epiglottitis, Infectious epiglottitis, Thumb sign

## Abstract

**Background:**

E-cigarette use, or vaping, is an alternative nicotine delivery system that is becoming increasingly prevalent in adolescents and young adults. There is currently a lack of comprehensive research on the adverse effects of vaping on the upper airway.

Acute epiglottitis is a potentially life-threatening condition that can lead to airway obstruction. It is commonly caused by bacterial infections such as streptococci, staphylococcus, and Moraxella. Adult patients with acute epiglottitis mainly present with odynophagia, dysphagia, and respiratory difficulties. The diagnosis of epiglottitis is made by direct laryngoscopy, and the mainstay of treatment is antibiotics.

Bozella et al. (2020) reported a case of subacute non-infectious epiglottitis associated with e-cigarette use in a pediatric patient (Pediatrics 145(3), 2020). Here we present a case of acute epiglottitis in a healthy young adult after vaping, with a negative infectious workup. To our knowledge, there has been no such reported case of epiglottitis associated with e-cigarette use in an adult patient.

**Case description:**

A previously healthy 29-year-old male with daily e-cigarette use presented to the emergency department with a severe sore throat, dysphagia, mild hoarseness, and shortness of breath, especially when lying supine. A lateral neck soft tissue radiograph revealed a thickened epiglottis with a thumb sign. Direct bedside laryngoscopy showed a swollen epiglottis, partially obstructing the supraglottic region confirming the diagnosis of acute epiglottitis. Throat and nasal swabs were negative for streptococcus and COVID-19 infection, respectively. The patient’s condition improved significantly after receiving intravenous Dexamethasone and antibiotics for 2 days. Repeat laryngoscopy showed the resolution of epiglottis swelling, and subjective symptoms had resolved entirely 2 weeks following the start of the treatment.

**Conclusions:**

Although bacterial infections usually cause acute epiglottitis, this case presents the second report of this condition associated with vaping with negative microbiological investigations. Therefore, we recommend that physicians consider non-infectious causes such as vaping in their differential diagnosis for patients with acute and subacute epiglottitis. More research is warranted on the utility of antibiotics in treating vaping-induced epiglottitis.

## Introduction

Electronic cigarette (e-cigarette) use or vaping is an alternative nicotine delivery system that has been promoted as a safer option to conventional smoking. E-cigarettes can be purchased prefilled or as reusable cartilages containing a mixture of flavours and various concentrations of nicotine, making them accessible and popular among teenagers and young adults. In 2018, it was estimated that 13 million Americans were active e-cigarette users [1]. There have been several reports of developing vaping-associated lung injury and atypical pneumonia in e-cigarette users [2–5], promoting researchers focusing on the adverse effects of vaping on the respiratory system. However, there is still a lack of comprehensive understanding of the effects of vaping on the upper respiratory tract.

E-cigarette smoking produces a vaporized liquid that passes through the pharynx, larynx, and vocal cords before reaching the lungs. This aerosol adheres to upper airway surfaces and can pose physiological changes to oropharyngeal health. In a recent in-vitro study, Lungova et al. showed epithelial erosion and disruption of the innate immune response 1 week after exposure to e-cigarette vapour extract. They further demonstrated epithelial and basal cell hyperplasia and membrane thickening [6].

Here we present a case of acute epiglottitis in an otherwise healthy young adult who was an active e-cigarette user. Considering the increasing prevalence of e-cigarette use in teenagers and young adults, we aim to highlight some of the unknown health risks associated with vaping. Further, we propose that primary practitioners and emergency physicians consider acute epiglottitis in their differential diagnosis for active e-cigarette users with acute or subacute dysphagia and odynophagia.

## Case presentation

A previously healthy 29-year-old male with no medical history of respiratory illness, recurrent throat infections, or hospital admissions presented to the emergency department with a 3-day history of progressively worsening sore throat, odynophagia, and dysphagia. On presentation day, he started experiencing mild hoarseness and shortness of breath, especially when lying supine, which prompted seeking medical attention. On further questioning, the patient was not taking any prescribed medications and had no known drug allergies. He reported continuous e-cigarette use starting 2 days prior to the development of symptoms, minimal alcohol intake, and no use of recreational drugs.

**Fig. 1:**
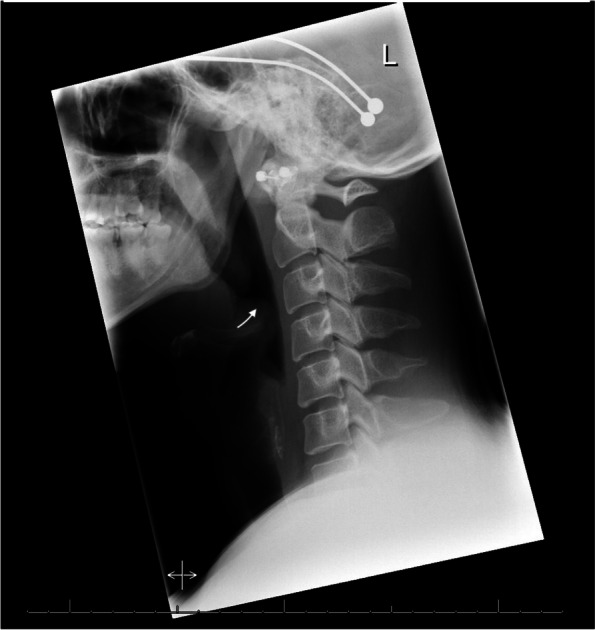
Lateral neck soft tissue radiograph: poorly defined and thickened epiglottis. The arrow points to the thumb sign which is commonly associated with acute epiglottitis

The patient received intravenous Dexamethasone In the emergency department, which immediately improved his shortness of breath. A throat culture was negative for group A, B, and C Streptococcus. COVID-19 was ruled out with a PCR nasal swab. The total peripheral white blood cell count was mildly elevated, but the rest of the renal and metabolic panels were normal. A lateral soft tissue radiograph of the neck showed a poorly defined and thickened epiglottis with a classic thumb sign, indicating acute epiglottitis (Fig. 1).

**Fig. 2:**
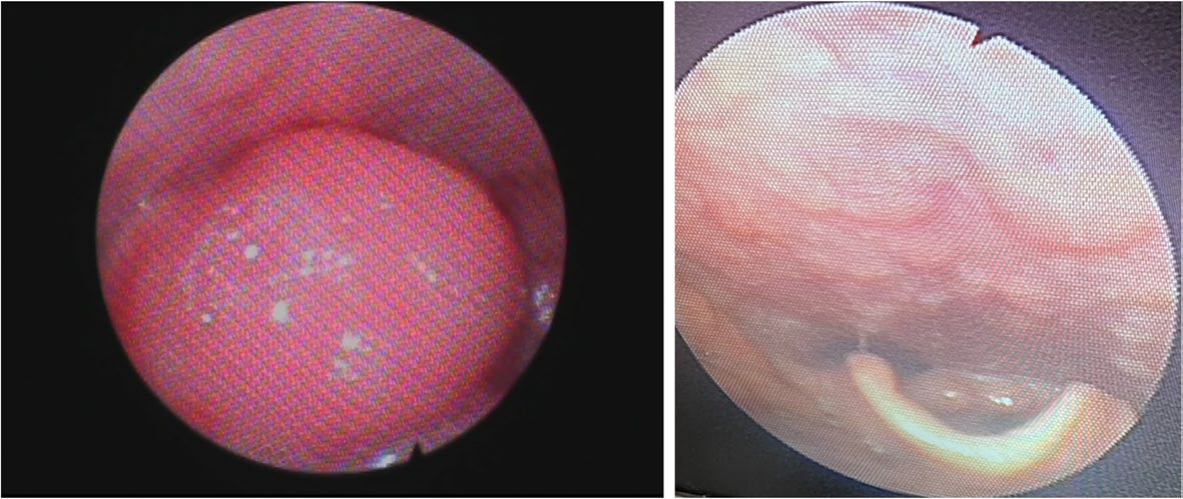
Direct laryngoscope examination of the epiglottis. **A** On initial presentation: the epiglottis appears swollen, erythematous, and is occluding the supraglottic region. **B** Post-therapy: normal appearing glottis, epiglottis, and larynx

Considering his respiratory concerns, the Otolaryngology team was consulted. On examination, the patient was afebrile and was not experiencing trismus or stridor. Direct bedside laryngoscopy showed an enlarged, erythematous, and swollen epiglottis, partially obstructing the supraglottic region (Fig. 2a). Arytenoids were noted to be edematous bilaterally with normal vocal cord mobility.

The patient was admitted to the hospital for airway monitoring and was started on intravenous Ceftriaxone and oral Metronidazole. He was also given another dose of intravenous Dexamethasone and was asked to stop using his e-cigarette.

The patient’s condition improved significantly after 2 days, and a repeat laryngoscopy showed resolution of the swelling in the epiglottis (Fig. 2b). The patient was switched to oral Amoxicillin for 10 days and was discharged from the hospital.

## Discussion and conclusions

Acute epiglottitis is a potentially life-threatening condition, often associated with an infection, which can lead to airway obstruction. It was historically considered a pediatric disease caused by Haemophilus influenza infection, but the incidence has dramatically declined in pediatrics after the introduction of the conjugate vaccines. There have been reports of a shift in etiology and epidemiology towards younger adults. Other more common etiologies are streptococci, staphylococcus, and Moraxella infections.

Adult patients with acute epiglottitis mainly present with odynophagia, dysphagia, drooling, and respiratory difficulties [7]. Direct laryngoscopy often shows a swollen, inflamed epiglottis, and soft tissue neck radiographs can often confirm an enlarged epiglottis [7, 8]. Acute epiglottitis is a clinical diagnosis, and direct visualization of the epiglottis remains the gold standard for diagnosis [9]. The mainstay of epiglottitis management is rapid airway assessment and maintenance, supplemental oxygen, and empirical antibiotic therapy [10]. Bronchodilators and parenteral glucocorticoids have also been suggested as additional therapies in severe cases [11].

E-cigarettes or vape pens are marketed as a healthier alternative to conventional smoking tobacco products. E-cigarette users can adjust the amount of nicotine delivered by each device; therefore, they can sometimes be used as an aid for smoking cessation and relieving withdrawal symptoms [12]. E-cigarettes are becoming increasingly prevalent in the adolescent and young adult population [13]. In recent years, a growing body of evidence has described the adverse effects and toxicity of e-cigarette use. In addition, there has been a report of microbial contamination in the E-cigarettes sold in the USA [14].

The biological and physiological effects of e-cigarettes on the oropharyngeal mucosa have been recently investigated. The oxidative stress causes increased production of inflammatory cytokines and decreased activity of innate immune cells [15]. Oral mucosal lesions such as hairy tongue and nicotine stomatitis are also more prevalent in e-cigarette users [16].

Bozella et al. (2020) reported a case of non-infectious acute epiglottitis in an adolescent female patient [17]. Similar to our case, the patient had presented to the emergency department with severe dysphagia and acute respiratory distress. However, their patient’s condition was more severe, and she needed a prolonged admission to the hospital and was treated with systemic antibiotics and Dexamethasone. After ruling out infectious etiologies, they hypothesized a direct chemical and thermal injury from vaping as the main cause of her epiglottitis.

This case presents the second report of acute epiglottitis directly associated with e-cigarettes with no evidence of infection. We cannot completely rule out the possibility of an infectious cause, and patients in both cases were treated with antibiotics. However, we also speculate that a primary direct thermal injury from the vaporized liquid promoted an inflammatory reaction in the supraglottic region, causing oxidative stress and epiglottis swelling. In addition, the oxidative stress causes increased viral replication and decreased antimicrobial activity of neutrophils and macrophages, resulting in immunosuppression and likely a secondary infection [15]. Moreover, antimicrobial and heavy metal contamination in the vape product could also play a role in producing this inflammatory reaction [14]. We recommend that physicians consider non-infectious primary causes such as vaping in their differential diagnosis for patients with acute and subacute epiglottitis. Future research is warranted on the utility of antibiotics in treating such patients with negative infectious workup.

## Data Availability

Data sharing is not applicable to this article as no datasets were generated or analyzed during the current study.

## Abbreviation

E-cigarettes: Electronic cigarettes
